# Factors associated with the specialty choice of Korean medical students: a cross-sectional survey

**DOI:** 10.1186/s12960-016-0141-8

**Published:** 2016-07-22

**Authors:** Yeon-Yong Kim, Un-Na Kim, Yon Su Kim, Jin-Seok Lee

**Affiliations:** Department of Health Policy and Management, Seoul National University College of Medicine, 110-799 103 Daehak-ro, Jongno-gu, Seoul, Republic of Korea; Department of Internal Medicine, Seoul National University College of Medicine, 110-799 103 Daehak-ro, Jongno-gu, Seoul, Republic of Korea; Institute of Health Policy and Management, Medical Research Center, Seoul National University, Seoul, Republic of Korea

**Keywords:** Health manpower, Medical education, Medical specialties, Specialty choice

## Abstract

**Background:**

An imbalance of physician supply by medical specialty has been observed in most countries. In Korea, there is a greater tendency to avoid surgical specialties and specialty choices in nonclinical medicine, such as the basic science of medicine. In this study, we identified factors affecting the specialty choice of physicians in order to provide a basis for policies to address this problem.

**Methods:**

We used the results of a 2013 nationwide survey of 12 709 medical students (82.7 % responded) to analyze the data of 9499 students after excluding missing data. Descriptive analyses of all students’ specialty choice were performed. Logistic regression was performed by selecting gender, age, grade level, type of medical school, hometown, and the location of the medical school as the independent variables. Medical specialty was the dependent variable. The dependent variable, or specialty of medicine, was categorized into three groups: nonclinical/clinical medicine, surgical-medical specialty, and controllable lifestyle specialty.

**Results:**

The order of preferred medical specialties was internal medicine, psychiatry, and pediatrics; for surgical specialties, the order was orthopedic surgery, general surgery, and ophthalmology. Medical specialties were most favored by women and students in the third (men) and second (women) year of the medical program, whereas surgical specialties were most preferred by men and students in the first year of the program. Students in the third year mostly favored nonclinical medicine. Medical college students had a stronger preference for nonclinical medicine (odds ratio [OR] 1.625, 95 % confidence interval [CI] 1.139–2.318) than graduate medical school students. Surgical specialties were more favored by men (OR 2.537, 95 % CI 2.296–2.804) than by women. However, they were favored less by medical college students (OR 0.885, 95 % CI 0.790–0.991) than by graduate medical school students and by medical students in metropolitan areas (OR 0.892, 95 % CI 0.806–0.988) than by medical students in nonmetropolitan areas. A controllable lifestyle specialty was less favored by men (OR 0.802, 95 % CI 0.730–0.881) than by women.

**Conclusions:**

Based on these results, we can evaluate the effectiveness of the government’s educational policies for solving the imbalance of physician supply and provide empirical evidence to understand and solve this problem.

## Background

The imbalance of the supply of physicians has been reported among different specialties and regions and between the public and private sectors. The disparity has been an important issue in most countries [1, 2]. In Korea, the supply imbalance among different specialties is problematic since the percentage of specialists is high among physicians (approximately 73 %) [3] and there is a large income gap among the specialties [4]. As a result, some specialties consistently have a short supply of physicians because of a lack of applicants relative to the number of openings, whereas for other specialties, it takes 2 or 3 years for some medical students to be admitted to the specialty due to the fierce competition.

In Korea, nonclinical medicine, such as the basic science of medicine, and certain surgical specialties are traditionally avoided for economic reasons. As the majority of healthcare institutions are private, economic factors have an important role in hospital management in Korea. Other than the economic aspects, the basic science of medicine is known to be avoided because of the instability of the position [5], and surgical specialties are avoided due to the high demand of physical intensity and stress [6]. Although it is impossible to measure the stability and stress of these positions, they are factors that significantly affect specialty choice [7] and downgrade lifetime utility [8].

Specialty choice is also influenced by the curriculum. Courses on the basic science of medicine are primarily taught in the lower grade of medical education, while students in the upper grade mostly learn clinical medicine, which eventually leads to their decreased interest in the basic science of medicine [9]. Educational programs for physicians also play an important role. Korea’s physician training system has been a 6-year undergraduate program in the medical college since 1946; however, a 4-year graduate program in the medical school, accepting graduates from different majors, was introduced in 2006 [10]. These two types of medical programs have similarities in their curricula but differences in the students’ experiences. The purpose of employing this system was based on the expectation that allowing the entrance of graduates who majored in diverse fields of study would enable diverse specialty choices other than clinical medicine [11, 12]. It was also expected that most of students who had majored in basic science before entering the graduate program of medical school would tend to choose specialties in nonclinical medicine in the hope of further developing their major studied at the undergraduate level [11]. Currently, both the 6-year medical college and 4-year graduate medical school programs coexist in Korea.

Although the problem of an imbalance of specialty choices is serious, studies on this subject have been limited in Korea [9, 13, 14]. In addition, doubts have been raised about whether the policy for solving the imbalance has achieved its goal, and there have not been enough studies on this matter. There has not yet been a proper evaluation of the representative policy, introducing a graduate medical school, which aimed to increase researchers in the basic science of medicine. In contrast, there is a substantial amount of relevant research, including surveys of the desired career paths of medical students, as well as matching programs in the United States and Japan [15–17].

In this study, we aimed to analyze factors affecting the specialty choices of medical students, based on the results of a survey taken by all medical students in Korea in 2013. In particular, the analysis focused on the factors affecting the choice of nonclinical medicine, surgical specialties, and controllable lifestyle specialties. This classification has been adopted in previous studies [9, 13–17]. Based on this study, we aimed to identify factors affecting students’ specialty choices and provide evidence for related policies to solve the imbalance in the physician supply.

## Methods

### Participants

The total number of participants was 12 709; 10 514 persons participated in the survey, yielding a response rate of 82.7 %. Excluding missing data, analyses were performed on survey data from 9499 respondents.

### Design and survey

This study used a cross-sectional design. A nationwide survey was distributed to medical college/graduate medical school students by the Ministry of Health and Welfare in 2013. The questionnaire included students’ school, grade, year of admission, gender, hometown, and desired medical specialty in the future.

### Variables

Gender, age, grade, type of medical school, hometown, and location of medical school were selected as independent variables. Type of medical school was divided into medical college and graduate medical school. Hometown was determined by the participant’s high school alma mater and classified as metropolitan (Seoul, Gyeonggi, and Incheon) or nonmetropolitan. The location of the medical school was also classified as metropolitan or nonmetropolitan. Grade was defined as the first, second, third, and fourth years of medical education. To test for the presence of multicollinearity, correlation analyses were performed with the independent variables, and it was confirmed that none of the variables had correlation coefficients above 0.8 for their relationships with any of the other variables (the correlation coefficient between type of medical school and age was the highest at 0.52).

The desired medical specialty for training was selected as the dependent variable. A total of 27 specialties was divided based on three criteria. The first criterion was whether the specialty belonged to clinical medicine or nonclinical medicine. Nonclinical medicine included pathology, preventive medicine, and the basic science of medicine, whereas clinical medicine included the remaining subspecialties (internal medicine, neurology, psychiatry, pediatrics, dermatology, therapeutic radiology, rehabilitative medicine, family medicine, nuclear medicine, emergency medicine, tuberculosis, occupational and environmental medicine, general surgery, orthopedic surgery, neurosurgery, plastic surgery, cardiothoracic surgery, obstetrics-gynecology, ophthalmology, otolaryngology, urology, anesthesiology, diagnostic radiology, and clinical pathology). The second criterion distinguished the medical and surgical specialties within clinical medicine. The surgical specialties included general surgery, orthopedic surgery, neurosurgery, plastic surgery, cardiothoracic surgery, obstetrics-gynecology, ophthalmology, otolaryngology, and urology. The remaining areas of clinical medicine were categorized as a medical specialty. The third criterion was whether the specialty offered a controllable lifestyle. Controllable lifestyle specialties were defined two ways and analyzed separately: specialties previously identified as offering a controllable lifestyle in a US-based study [16] and specialties in which a majority of practitioners were self-employed in Korea. These classifications are not well established in the literature; therefore, we did not attempt to investigate the data further with subgroup analyses. For the former definition, the specialties offering a controllable lifestyle included anesthesiology, dermatology, emergency medicine, neurology, ophthalmology, otolaryngology, pathology, psychiatry, and diagnostic radiology. Specialties with noncontrollable lifestyles in a US-based study included internal medicine, family medicine, pediatrics, obstetrics-gynecology, orthopedic surgery, general surgery, and urology. For the latter definition, the specialties with more than 50 % of self-employed specialists [18], such as dermatology, otolaryngology, plastic surgery, ophthalmology, urology, pediatrics, family medicine, obstetrics-gynecology, and tuberculosis, were included in the controllable lifestyle specialty category.

### Data analysis

A descriptive analysis of the clinical/nonclinical specialties and an analysis of the controllable/noncontrollable lifestyle specialties in the self-employed specialties were performed on data from 9499 persons. An analysis of the surgical/nonsurgical specialties was performed on data from 9313 persons, excluding those who desired nonclinical medicine. An analysis of the controllable/noncontrollable lifestyle specialties in a US-based study was conducted on the data from 8259 persons, excluding those who desired specialties that were not included in our categories.

A descriptive analysis was performed on the specialty choices of all of the students, and logistic regression was conducted to find the factors influencing specialty choice. In the analyses of the nonclinical medicine and surgical specialties, a gender subgroup analysis was performed. SPSS 22.0 was used for the statistical analyses, and the significance level was set at *P* < 0.05. This study received an exemption from the Seoul National University Hospital Institutional Review Board (IRB No. 1506-132-683).

## Results

### General characteristics

The general characteristics of the study participants are presented in Table 1. There were more men (60.9 %) than women (39.1 %), and the number of students from nonmetropolitan areas (57.6 %) was higher than that from metropolitan areas (42.4 %). There were more graduate medical school students (60.4 %) than medical college students (39.6 %), and the number of students in nonmetropolitan medical schools (66.8 %) was higher than that in metropolitan medical schools (33.2 %). The average age was 25.5 years old.

**Table 1:** General characteristics (*N* = 9499)

	Grade (year)	1st		2nd		3rd		4th		Total		*P* value
			%		%		%		%		%	
Gender	Men	1 747	64.8	1 473	60.7	1 276	61.0	1 288	56.3	5 784	60.9	<0.001
	Women	947	35.2	954	39.3	816	39.0	998	43.7	3 715	39.1	
Hometown	Metropolitan	1 160	43.1	1 028	42.4	860	41.1	981	42.9	4 029	42.4	0.541
	Nonmetropolitan	1 534	56.9	1 399	57.6	1 232	58.9	1 305	57.1	5 470	57.6	
Type of medical school	Medical college	1 137	42.2	979	40.3	839	40.1	808	35.3	3 763	39.6	<0.001
	Graduate medical school	1 557	57.8	1 448	59.7	1 253	59.9	1 478	64.7	5 736	60.4	
Location of medical school	Metropolitan	871	32.3	847	34.9	547	26.1	891	39.0	3 156	33.2	<0.001
	Nonmetropolitan	1 823	67.7	1 580	65.1	1 545	73.9	1 395	61.0	6 343	66.8	
Age (mean, SD)		23.82 (2.94)		25.00 (2.99)		26.34 (3.14)		27.37 (3.05)		25.53 (3.32)		<0.001

Chi-square test was performed for gender, hometown, type of medical school, and location of medical school. Analysis of variance (ANOVA) was performed for age

### Descriptive analysis

In the descriptive analysis of the desired specialties of all the students, the preference among the medical specialties was highest for internal medicine (29.8 %), followed by psychiatry (7.8 %), and pediatrics (6.8 %), whereas the preference among the surgical specialties was highest for orthopedic surgery (9.0 %), followed by general surgery (6.7 %), and ophthalmology (3.7 %) (Table 2). The percentage of students who preferred medical specialties was the highest (67.6 %), with the percentages of students choosing surgical specialties and nonclinical medicine being 30.4 % and 2.0 %, respectively.

**Table 2:** Specialty preferences of medical students (*N* = 9499)

Grade/gender specialty			1st		2nd		3rd		4th		Total
			Men	Women	Men	Women	Men	Women	Men	Women	
Clinical	Medical	Internal medicine	478 (27.4)*	283 (29.9)*	461 (31.3)*	326 (34.2)*	368 (28.8)*	282 (34.6)*	350 (27.2)*	287 (28.8)*	2 835 (29.8)
		Psychiatry	144 (8.2)*	98 (10.3)*	94 (6.4)	84 (8.8)	82 (6.4)	68 (8.3)	83 (6.4)	91 (9.1)	744 (7.8)
		Pediatrics	51 (2.9)*	101 (10.7)*	45 (3.1)*	105 (11.0)*	58 (4.5)*	110 (13.5)*	50 (3.9)*	123 (12.3)*	643 (6.8)
		Dermatology	79 (4.5)	59 (6.2)	45 (3.1)	65 (6.8)	48 (3.8)	35 (4.3)	39 (3.0)	50 (5.0)	420 (4.4)
		Neurology	52 (3.0)	48 (5.1)	56 (3.8)	50 (5.2)	48 (3.8)	39 (4.8)	47 (3.6)	38 (3.8)	378 (4.0)
		Rehabilitative medicine	75 (4.3)*	36 (3.8)*	57 (3.9)*	24 (2.5)*	48 (3.8)*	14 (1.7)*	51 (4.0)*	32 (3.2)*	337 (3.5)
		Diagnostic radiology	57 (3.3)*	32 (3.4)*	37 (2.5)	43 (4.5)	35 (2.7)	26 (3.2)	33 (2.6)	46 (4.6)	309 (3.3)
		Anesthesiology	37 (2.1)	30 (3.2)	39 (2.6)	28 (2.9)	35 (2.7)*	20 (2.5)*	43 (3.3)	42 (4.2)	274 (2.9)
		Family medicine	22 (1.3)	20 (2.1)	23 (1.6)	33 (3.5)	23 (1.8)	23 (2.8)	24 (1.9)	37 (3.7)	205 (2.2)
		Emergency medicine	22 (1.3)*	8 (0.8)*	24 (1.6)*	8 (0.8)*	21 (1.6)	12 (1.5)	18 (1.4)*	8 (0.8)*	121 (1.3)
		Clinical pathology	6 (0.3)	7 (0.7)	3 (0.2)	4 (0.4)	6 (0.5)	5 (0.6)	2 (0.2)*	13 (1.3)*	46 (0.5)
		Therapeutic radiology	8 (0.5)*	1 (0.1)*	6 (0.4)	1 (0.1)	3 (0.2)	5 (0.6)	12 (0.9)	5 (0.5)	41 (0.4)
		Occupational and environmental medicine	5 (0.3)	0 (0.0)	4 (0.3)	0 (0.0)	8 (0.6)	3 (0.4)	12 (0.9)	5 (0.5)	37 (0.4)
		Nuclear medicine	3 (0.2)	0 (0.0)	3 (0.2)	0 (0.0)	7 (0.5)	0 (0.0)	3 (0.2)	0 (0.0)	16 (0.2)
		Tuberculosis	1 (0.1)	0 (0.0)	2 (0.1)	1 (0.1)	1 (0.1)	0 (0.0)	9 (0.7)*	1 (0.1)*	15 (0.2)
		Subtotal	1 040 (59.5)*	723 (76.3)*	899 (61.0)*	772 (80.9)*	791 (62.0)*	642 (78.7)*	776 (60.2)*	778 (78.0)*	6 421 (67.6)
	Surgical	Orthopedic surgery	257 (14.7)*	14 (1.5)*	222 (15.1)*	4 (0.4)*	169 (13.2)*	7 (0.9)*	174 (13.5)*	6 (0.6)*	853 (9.0)
		General surgery	129 (7.4)*	67 (7.1)*	102 (6.9)*	46 (4.8)*	109 (8.5)*	50 (6.1)*	79 (6.1)*	51 (5.1)*	633 (6.7)
		Ophthalmology	83 (4.8)*	50 (5.3)*	57 (3.9)*	27 (2.8)*	21 (1.6)	20 (2.5)	52 (4.0)	41 (4.1)	351 (3.7)
		Neurosurgery	87 (5.0)*	20 (2.1)*	61 (4.1)*	21 (2.2)*	54 (4.2)*	5 (0.6)*	53 (4.1)*	7 (0.7)*	308 (3.2)
		Otolaryngology	50 (2.9)*	16 (1.7)*	37 (2.5)*	18 (1.9)*	35 (2.7)*	13 (1.6)*	41 (3.2)	28 (2.8)	238 (2.5)
		Plastic surgery	32 (1.8)*	2 (0.2)*	34 (2.3)*	14 (1.5)*	36 (2.8)*	12 (1.5)*	63 (4.9)*	16 (1.6)*	209 (2.2)
		Obstetrics-gynecology	8 (0.5)	15 (1.6)	3 (0.2)*	31 (3.2)*	9 (0.7)*	38 (4.7)*	8 (0.6)*	45 (4.5)*	157 (1.7)
		Cardiothoracic surgery	27 (1.5)*	14 (1.5)*	26 (1.8)*	6 (0.6)*	20 (1.6)*	6 (0.7)*	12 (0.9)	10 (1.0)	121 (1.3)
		Urology	3 (0.2)	1 (0.1)	4 (0.3)	1 (0.1)	6 (0.5)	0 (0.0)	5 (0.4)	2 (0.2)	22 (0.2)
		Subtotal	676 (38.7)*	199 (21.0)*	546 (37.1)*	168 (17.6)*	459 (36.0)*	151 (18.5)*	487 (37.8)*	206 (20.6)*	2 892 (30.4)
Nonclinical		Pathology	10 (0.6)	8 (0.8)	6 (0.4)	7 (0.7)	12 (0.9)	16 (2.0)	9 (0.7)	8 (0.8)	76 (0.8)
		Basic science of medicine	17 (1.0)	9 (1.0)	16 (1.1)*	3 (0.3)*	9 (0.7)*	2 (0.2)*	11 (0.9)*	3 (0.3)*	70 (0.7)
		Preventive medicine	4 (0.2)	8 (0.8)	6 (0.4)	4 (0.4)	5 (0.4)	5 (0.6)	5 (0.4)	3 (0.3)	40 (0.4)
		Subtotal	31 (1.8)	25 (2.6)	28 (1.9)	14 (1.5)	26 (2.0)	23 (2.8)	25 (1.9)	14 (1.4)	186 (2.0)

*P < 0.05, chi-square test was performed for specialty (grouped by grade and gender)

In all grades, medical specialties were preferred more by women than men, except rehabilitative medicine, emergency medicine, occupational and environmental medicine, nuclear medicine, and tuberculosis, which were favored more by men. The preference for the medical specialties by grade was highest in the third year in the men (62.0 %) and the second year in the women (80.9 %) and was lowest in the first year in both the men (59.5 %) and the women (76.3 %). However, psychiatry and rehabilitative medicine were most preferred by the first year students, whereas anesthesiology, family medicine, therapeutic radiology, and occupational and environmental medicine were most preferred by the fourth year students in both men and women.

Surgical specialties were favored more by men than women in all grades, except obstetrics-gynecology, which was preferred by women. The highest preference for surgical specialties was found in the first year in both men (38.7 %) and women (21.0%) and the lowest preference was found in the third year in men (36.0 %) and second year in women (17.6 %). However, general surgery was most preferred by men in the third year (8.5 %), and otolaryngology and plastic surgery were most preferred by both the men and women in the fourth year. For nonclinical medicine, the highest preference was found in both men (2.0 %) and women (2.8 %) in the third year and the lowest preference was found in the first year men (1.8 %) and fourth year women (1.4 %).

### Logistic regression results

The number of students hoping to specialize in nonclinical medicine was 186 out of the 9499 (2.0 %); among these, 110 were men and 76 were women. The results of the logistic regression for nonclinical medicine are presented in Table 3. The medical college students had a greater desire to specialize in nonclinical medicine than the graduate medical school students did, and the difference was significant. Other variables were not statistically significant. In the subgroup analysis by gender, men were more likely to favor nonclinical medicine when they were medical college students rather than graduate medical school students and if they were born in a metropolitan area than in a nonmetropolitan area. These differences were significant. In women, there was a significant difference by grade, with the first year students showing a higher preference for nonclinical medicine. The difference between medical college and graduate medical school was not significant.

**Table 3:** Logistic regression for factors associated with nonclinical medicine specialty *N* = 9499)

		All	Subgroup analysis	
		aOR (95 % CI)	Men	Women
			aOR (95 % CI)	aOR (95 % CI)
Men (ref: women)		0.851 (0.628–1.152)		
Age		1.052 (0.998–1.110)	1.038 (0.971–1.110)	1.098 (0.999–1.207)
Grade	1st	Reference	Reference	Reference
	2nd	0.780 (0.518–1.174)	1.040 (0.617–1.753)	0.488* (0.249–0.956)
	3rd	1.011 (0.671–1.522)	1.047 (0.603–1.820)	0.857 (0.459–1.597)
	4th	0.689 (0.438–1.084)	0.964 (0.541–1.717)	0.374* (0.176–0.793)
Medical college (ref: graduate medical school)		1.625* (1.139–2.318)	1.768* (1.114–2.807)	1.484 (0.823–2.677)
Metropolitan hometown (ref: nonmetropolitan)		1.161 (0.857–1.573)	1.513* (1.023–2.240)	0.783 (0.481–1.274)
Metropolitan school (ref: nonmetropolitan)		1.183 (0.858–1.631)	1.198 (0.794–1.808)	1.189 (0.709–1.993)

*P < 0.05

The number of students desiring a surgical specialty was 2892 out of 9313, after excluding students who wished to specialize in nonclinical medicine. Among them, 2168 were men and 724 were women. The results of the logistic regression for factors affecting the choice of surgical specialties are presented in Table 4. A preference for surgical specialties was significantly higher in men than in women and in graduate medical school students than in medical college students. Other variables were not statistically significant. The subgroup analysis by gender revealed that, among the men, surgical specialties were preferred by the younger students, by the graduate medical school students over medical college students, and by students at medical schools located in nonmetropolitan rather than metropolitan areas. For women, those in the first year preferred surgical specialties. Other factors were not statistically significant.

**Table 4:** Logistic regression for factors associated with surgical specialty preference (*N* = 9313)

		All	Subgroup analysis	
		aOR (95 % CI)	Men	Women
			aOR (95 % CI)	aOR (95 % CI)
Men (ref: women)		2.537* (2.296–2.804)		
Age		0.985 (0.968–1.003)	0.974* (0.954–0.995)	1.024 (0.986–1.064)
Grade	1st	Reference	Reference	Reference
	2nd	0.907 (0.802–1.027)	0.967 (0.836–1.119)	0.768* (0.608–0.970)
	3rd	0.909 (0.795–1.040)	0.949 (0.809–1.113)	0.805 (0.625–1.038)
	4th	1.022 (0.890–1.173)	1.065 (0.903–1.256)	0.876 (0.677–1.135)
Medical college (ref: graduate medical school)		0.885* (0.790–0.991)	0.849* (0.743–0.970)	1.009 (0.810–1.256)
Metropolitan hometown (ref: nonmetropolitan)		1.031 (0.938–1.134)	1.055 (0.941–1.182)	0.970 (0.815–1.154)
Metropolitan school (ref: nonmetropolitan)		0.892* (0.806–0.988)	0.840* (0.743–0.949)	1.032 (0.858–1.241)

*P < 0.05

In the analysis of controllable lifestyle specialties, 2911 of the 8259 students (35.2 %) desired specialties with a controllable lifestyle, after excluding those who desired specialties not included in the existing controllable/noncontrollable classification (1240 students). The results of the logistic regression for factors affecting specialty choice for controllable lifestyle specialties showed a significantly higher preference in women over men, first year over second and third years, and medical college students over graduate medical school students (Table 5). For further comparison, the analysis was performed after categorizing the specialties with more than 50 % of self-employed specialists as controllable lifestyle specialties (Table 5). The results showed that women, older students, students originally from a metropolitan area, students currently attending a nonmetropolitan medical school, and fourth year students (reference first year) had a stronger preference for a controllable lifestyle specialty.

**Table 5:** Logistic regression for factors associated with controllable lifestyle specialty preference

		Controllable lifestyle	Self-employed specialty
		[*N* = 8259]	[*N* = 9499]
		aOR (95 % CI)	aOR (95 % CI)
Men (ref: women)		0.802* (0.730–0.881)	0.519* (0.470–0.573)
Age		0.992 (0.974–1.011)	1.021* (1.002–1.041)
Grade	1st	Reference	Reference
	2nd	0.848* (0.749–0.961)	0.982 (0.858–1.124)
	3rd	0.779* (0.679–0.894)	0.987 (0.853–1.142)
	4th	0.965 (0.839–1.110)	1.229* (1.061–1.424)
Medical college (ref: graduate medical school)		1.198* (1.067–1.344)	1.118 (0.990–1.263)
Metropolitan hometown (ref: nonmetropolitan)		0.992 (0.901–1.092)	1.143* (1.033–1.264)
Metropolitan school (ref: nonmetropolitan)		1.030 (0.930–1.140)	0.779* (0.698–0.870)

*P < 0.05

## Discussion

In this study, we examined the desired specialties of medical students and the factors affecting specialty choice. We found that medical specialties were most favored by women and middle grade students, whereas surgical specialties were most preferred by men and lower grade students. Nonclinical medicine was most favored by lower grade students. The statistically significant results of the logistic regression can be summarized as follows. Nonclinical medicine was more preferred by medical college students than by graduate medical school students, by students from metropolitan areas among the men, and by lower grade students among the women. Surgical specialties were favored more by men than by women, by graduate medical school students than by medical college students, and by medical students from medical schools in nonmetropolitan areas than in metropolitan areas. The women favored the controllable lifestyle specialties more than the men did.

According to the classification of specialties by Organisation for Economic Cooperation and Development (OECD) health data [3], the proportion of specialists in Korea in 2013 was found to be 72.7 %, including 34.7 % in a medical specialty, 32.9 % in a surgical specialty, and 5.1% in other specialties. Compared to the United States and United Kingdom, the percentage of medical specialists in Korea appears to be similar, whereas the percentage of surgical specialists is higher [3]. However, in a comparison with the United States [19] at the level of individual specialties, Korea showed a relatively stronger preference for orthopedic surgery and neurosurgery rather than general surgery, and this seems attributable to the fact that the income levels of orthopedic surgeons and neurosurgeons are much higher than that of others [20, 21].

The present application status for specialties in 2015, which consists of applications made by upper grade students who participated in the survey in 2013, revealed that the order of preferred specialties was internal medicine, orthopedic surgery, family medicine, pediatrics, anesthesiology, diagnostic radiology, and psychiatry [22]. Although it is impossible to make direct comparisons, similar tendencies were observed in the results of our survey. However, certain specialties, such as family medicine, were ranked higher among the actual applications but lower in the survey, and other specialties showed the opposite pattern; therefore, further studies are needed to explain these differences.

Nonclinical medicine was favored more by medical college students than by graduate medical school students, which is contrary to the government initiative of utilizing graduate medical schools. In the United States, medical scientist training programs have been used to nurture basic scientists with a medical background, and they have been effective to a certain extent [23]. A similar program for nurturing basic scientists in medicine was employed in graduate medical school programs in Korea, but it was discontinued because of its lack of results [10]. According to this study, prioritizing policies for medical colleges rather than graduate medical schools, and to women rather than men, should be more effective. Grade-dependent patterns of preferences showed a clear difference among the women in this study; here, nonclinical medicine was preferred more by the students in the lower grade, which is when most of the courses in the basic science of medicine are taken. Therefore, the curriculum needs to be improved to enable students in the upper grade to experience the basic science of medicine as well. In particular, for the specialties requiring greater demands that are chosen least often by the students, the trends in specialty choice during the years as a medical student are more important [24]. Therefore, these implications should be addressed more seriously in the curriculum.

Surgical specialties were more favored by men, which is consistent with a previous study [13, 17], which found that graduate medical school students had a stronger preference for surgical specialties than medical college students did. Surgical specialties are a mixture of highly competitive (plastic surgery and orthopedic surgery) and noncompetitive subspecialties (cardiothoracic surgery and general surgery). We found that surgical specialties were preferred by men in both Korea and the United States. However, in the United States, some surgical subspecialties have become more appealing to women in recent years [17]. Therefore, routine surveys investigating trends in specialty preferences are needed. Yet, there was no difference in preferences between medical college students and graduate medical students according to the popularity of the subspecialty. Therefore, the trend appears to be caused by the broad category of features of the surgical specialty, rather than by noncompetitive subspecialties. However, in studies in the United States, MD-PhD students had a lower preference for surgical specialties, unlike those in Korea [25, 26]. The results are not easily compared with those of graduate medical schools in Korea, considering the environmental differences. It was found that nonmetropolitan medical students had a stronger preference for surgical specialties than metropolitan medical students, and it can be assumed that there are relatively more job opportunities in nonmetropolitan hospitals than in metropolitan hospitals, where physician manpower is saturated [27, 28].

Women were found to have a stronger preference for a controllable lifestyle specialty. The percentage of students (upper grade) selecting the specialties categorized as controllable lifestyle was higher in women (39.0 %) than in men (34.6 %), which is different from the results of a study by Lambert et al. [17], in which men had a stronger preference than women for a controllable lifestyle specialty (men 40.7 %, women 34.4 %). It is impossible to explain fully the discrepancy in specialty preference according to gender, but it is possible that a characteristic of the Korean culture, in which women have more responsibility for the housekeeping burden, could have had an effect. Specialties for which more than 50 % of the specialists went into clinical practice were favored by students who were women, older, from a metropolitan area, currently attending a nonmetropolitan medical school, and in their fourth year of studies. Both analyses found that women had a stronger preference for a controllable lifestyle specialty. Controllable lifestyle specialties were defined as those with less stress, more free time, a lower amount of work-related activity, and fewer demands. Considering a report [29] showing that self-employed doctors are more satisfied because they have autonomy, flexibility, skill utilization, and higher job security, we can also regard this distinction as an applicable standard for a controllable lifestyle specialty.

In order to solve the imbalances of physician supply, it is essential to monitor major determining factors, including factors influencing specialty choice [30]. Specialty choice varies according to the grade and the curriculum of the medical student. Therefore, the routine monitoring of students’ preferences should help track preference changes. Through this study, we were able to identify factors affecting specialty choice and provide evidence for solutions to the imbalances in physician specialties. The study is limited by the failure to include a variety of variables relevant to specialty choice. Individual factors (e.g., marital status, number of dependent children, student academic performance, and financial status/amount of student debt) and specialty-related factors (e.g., labor demands, remuneration, specialty prestige, and the length of training) might significantly influence specialty choice although they have not been fully examined in previous studies. However, the present study has significant implications as the sample included most of the medical students in Korea. Continuous research on factors affecting specialty choice based on this study will be required in the future.

## Conclusions

In this study, a descriptive analysis was performed on the choice of specialty and we were able to identify factors affecting students’ choices of nonclinical medicine, surgical specialties, and controllable lifestyle specialties. Nonclinical medicine was more favored by medical college students, and surgical specialties were more favored by men, graduate medical school students, and students from nonmetropolitan medical schools. Depending on the type of analysis (using the existing categorizations or the percentage of self-employed specialists), the factors influencing the choice of a controllable lifestyle specialty were different. However, in both approaches, women consistently showed a stronger preference for a controllable lifestyle specialty. Based on these results, we can evaluate the effectiveness of governmental policies to resolve the supply imbalance and provide theoretical evidence for a solution to it.
